# Probiotic co-administration attenuates developmental cafeteria diet–induced cellular stress and NLRP3 inflammasome signaling in the spleen

**DOI:** 10.3389/fnut.2026.1848057

**Published:** 2026-07-09

**Authors:** Aysun Inan Genc

**Affiliations:** Department of Biology, Kastamonu University, Kastamonu, Türkiye

**Keywords:** apoptosis, autophagy, cafeteria diet, developmental period, NLRP3 inflammasome, SCD Probiotics, spleen

## Abstract

**Background:**

Early life exposure to obesogenic diets is increasingly associated with persistent immunometabolic dysregulation. However, the effects of such dietary stress on splenic apoptosis, autophagy, and inflammasome signaling during the developmental period remain insufficiently characterized. This study investigated whether probiotic supplementation modulates cafeteria diet (Cd)-induced molecular alterations in splenic tissue during the post-weaning period.

**Methods:**

Twenty-one-day-old male Wistar rats were randomly assigned to four groups (*n* = 7/group): Control (Cnt), Cafeteria diet (Cd), SCD Probiotics (Prb), and Cafeteria diet plus probiotic (Cd+Prb). Interventions were maintained from postnatal day 21 to 56. Splenic apoptosis, autophagy, and inflammasome related markers were evaluated using RT-qPCR and immunohistochemistry.

**Results:**

Cafeteria diet exposure shifted the spleen toward a pro-apoptotic state, characterized by a relative decrease in BCL2 fold-change pattern and relative increases in BAK and Caspase-3 fold-change patterns, while protein analysis confirmed marked elevations in Caspase-3 and BAX (both *p* < 0.0001). Autophagy-related alterations included reduced ATG5 expression and marked p62 accumulation, consistent with impaired autophagic regulation, with corresponding protein-level differences for ATG5 and p62 (both *p* < 0.0001). Inflammasome-associated signaling was reflected by relative increases in NLRP3 and IL-18 fold-change patterns together with higher protein-level immunoreactivity, with strong protein-level significance (*p* < 0.0001). Probiotic administration exerted context-dependent effects on inflammasome-related gene expression, whereas probiotic co-administration attenuated several Cd-induced alterations, including partial normalization of BCL2 and IL-18 fold-change patterns and reduction of NLRP3 protein levels compared with the Cd group (*p* < 0.0001), although complete normalization was not achieved across all markers.

**Conclusion:**

Developmental exposure to a cafeteria diet was associated with alterations in splenic apoptotic, autophagy-associated, and inflammasome-related markers, consistent with immune dysregulation during the post-weaning period. Concurrent probiotic supplementation partially and context-dependently modulated several of these alterations.

## 1 Introduction

The developmental period constitutes a critical phase of immune and metabolic programming during which nutritional exposures can exert long-lasting effects on systemic homeostasis. Accumulating evidence indicates that early-life consumption of energy-dense, high-fat diets induces persistent immunometabolic remodeling characterized by chronic low-grade inflammation, altered cellular bioenergetics, and dysregulated stress signaling (1, 2). While the metabolic consequences of obesogenic diets have been extensively investigated in liver and adipose tissue, comparatively less attention has been directed toward secondary lymphoid organs such as the spleen. As a central immunological hub integrating innate and adaptive responses, the spleen reflects systemic inflammatory cues and circulating metabolic signals. Conceptual advances in immunometabolism emphasize that immune cell function is tightly governed by intracellular metabolic programs and by organism-level metabolic environments (3, 4). Moreover, chronic metabolic inflammation has been recognized as a systemic process involving coordinated immune–metabolic communication across tissues (5). In parallel, probiotic supplementation has been reported to modulate systemic inflammatory tone and metabolic endotoxemia in diet-induced obesity models, suggesting a potential role in shaping immunometabolic programming (6, 7). However, whether such immunometabolic remodeling extends to secondary lymphoid organs during critical developmental windows remains insufficiently defined.

At the cellular level, metabolic overload perturbs interconnected stress response pathways that determine immune cell fate. The intrinsic apoptotic cascade, governed by BCL-2 family dynamics and caspase activation, represents a primary mechanism by which lipid excess reshapes tissue integrity (8, 9). In parallel, autophagy functions as a metabolically sensitive quality-control system that preserves mitochondrial integrity and cellular viability under nutrient imbalance. Dysregulated autophagic flux has been implicated in metabolic inflammation and immune dysfunction (10, 11). Emerging experimental evidence further suggests that certain probiotic strains can attenuate caspase dependent apoptotic signaling and modulate autophagy related pathways under inflammatory and metabolic stress conditions (12). Importantly, apoptosis and autophagy form a tightly coordinated regulatory network whose imbalance under metabolic stress may determine splenic immune resilience or vulnerability.

Concurrently, inflammasome activation has emerged as a central node linking metabolic stress to inflammatory amplification. The NLRP3 inflammasome integrates mitochondrial dysfunction, redox imbalance, and lipid derived signals to promote IL-1 family cytokine maturation and systemic immune activation (13). Experimental studies indicate that probiotic administration can suppress NLRP3 inflammasome activation and reduce IL-1β production in models of metabolic and inflammatory stress (7). Modulation of microbiota immune interactions through probiotic supplementation has therefore gained attention as a strategy to attenuate diet-associated inflammatory signaling. However, whether developmental probiotic intervention modulates concurrent apoptosis, autophagy, and inflammasome related alterations in splenic tissue under obesogenic stress remains unclear. Therefore, this study examined whether developmental cafeteria diet exposure is associated with splenic immunometabolic dysregulation and whether probiotic co-administration attenuates these alterations.

Based on this framework, the present study was designed to determine whether developmental cafeteria diet exposure induces immunometabolic dysregulation in splenic tissue and whether concurrent probiotic co-administration mitigates apoptosis-associated, autophagy-related, and inflammasome mediated alterations. We hypothesized that early life obesogenic stress promotes coordinated activation of apoptotic and inflammasome pathways alongside impaired autophagic regulation, and that probiotic co-administration partially restores splenic immunometabolic homeostasis.

## 2 Materials and methods

### 2.1 Experimental design and animal care

Twenty-one-day-old male Wistar rats were used at the onset of the post-weaning developmental period. Following weaning (postnatal day 21), animals were randomly assigned to four experimental groups (*n* = 7 per group): (i) Control (Cnt), receiving standard laboratory rodent chow; (ii) Cafeteria diet (Cd), receiving standard chow plus a cafeteria-style high-fat diet; (iii) Probiotic (Prb), receiving standard chow supplemented with SCD Probiotics; and (iv) Cafeteria diet plus probiotic (CdPrb), receiving both the cafeteria diet and SCD Probiotics supplementation. Experimental interventions were maintained from postnatal day 21 to postnatal day 56.

SCD Probiotics (Essential Probiotics XI – 500 mL; H.S. Code: 2206.00.7000), a commercially available dietary supplement produced by a certified manufacturer, were administered via oral gavage at a daily dose of 1.5 mL containing 1 × 108 CFU, as previously described (14). The formulation comprised *Bacillus subtilis, Bifidobacterium bifidum, Bifidobacterium longum, Lactobacillus acidophilus, Lactobacillus bulgaricus, Lactobacillus casei, Lactobacillus fermentum, Lactobacillus plantarum, Lactococcus lactis, Saccharomyces cerevisiae*, and *Streptococcus thermophilus*. Animals had *ad libitum* access to standard chow throughout the experiment, and the cafeteria diet was provided as an additional energy-dense source in the relevant groups. The detailed composition of the cafeteria diet has been previously described in our earlier studies using the same experimental design and animal cohort (15), and this diet was provided in addition to standard *ad libitum* feeding. Throughout the experiment, the animals' weights, weekly food consumption, and measurements of the cafeteria diet content were recorded, as demonstrated in earlier investigations (15, 16).

At postnatal day 56, animals were euthanized by rapid decapitation using a guillotine, without prior anesthesia, in accordance with the approved animal ethics protocol. Spleen tissues were rapidly excised, snap-frozen on dry ice, and stored at −80 °C until molecular and histological analyses. For downstream procedures, tissues were homogenized under sterile conditions. Each spleen was finely minced using a sterile scalpel to ensure sample uniformity prior to processing. All qPCR analyses were performed on five randomly selected biological samples from each group, with duplicate technical measurements for each sample (15). All procedures were conducted in accordance with institutional guidelines for animal care and use and were approved by the Bingöl University Animal Experiments Local Ethics Committee (approval no: 2021/03; meeting date: 29.06.2021).

### 2.2 RNA isolation and cDNA synthesis

Total RNA extraction from splenic tissue samples was performed using the GeneAll Hybrid-R purification kit (Cat. No. 305-101, Korea) in accordance with the manufacturer's recommended protocol. RNA yield and purity were evaluated spectrophotometrically using a Multiskan GO microplate reader (Thermo Scientific, Waltham, MA, USA) integrated with NanoDrop QC SkanIt Software (version 4.1). RNA integrity and concentration values were confirmed prior to downstream applications.

Complementary DNA (cDNA) synthesis was subsequently carried out using the ProtoScript First Strand cDNA Synthesis Kit (SuScript cDNA Synthesis Kit, Cat. No. RT01A025, Turkey) following the manufacturer's instructions. Briefly, reverse transcription reactions included an initial denaturation step at 70 °C for 5 min, incubation at 42 °C for 60 min to allow reverse transcription, and final enzyme inactivation at 80 °C for 5 min. All thermal cycling steps were conducted in a SensoQuest Labcycler thermocycler (Thermoblock 96 Gold Plated Silver 012-103, Germany). The resulting cDNA products were stored at −20 °C until use in quantitative gene expression analyses (17).

### 2.3 Primer design and quantitative gene expression analysis

Gene-specific primers were designed using the Primer3 online platform (http://primer3.ut.ee/) to ensure optimal amplification efficiency and specificity. Quantitative real-time PCR (qPCR) assays were performed using the Rotor-Gene Q real-time PCR system (QIAGEN, Hilden, Germany) in combination with the SuScript 1-Step SYBR Green qPCR Kit (Catalog No: RT01A046, 200 reactions, Ankara, Turkey). Reactions were prepared in a total volume of 10 μL using 0.1 mL strip tubes. The amplification protocol consisted of an initial incubation at 50 °C for 2 min, followed by a pre-denaturation step at 95 °C for 1 min, and 40 amplification cycles comprising 95 °C for 10 s and 60 °C for 30 s. GAPDH was employed as the endogenous reference gene for normalization. qPCR CT values were converted to the relative mRNA expressions using the 2-ΔΔCT calculation method proposed by Livak and Schmittgen (18). The primer sequences utilized in this study are provided in Table 1.

**Table 1:** Sequences of specific primers.

Gene name	Elongation position	Sequence (5^′-3′^)	References
Apoptosis	
BCL2	Forward	GAGGATTGTGGCCTTCTTTG	Guo and Li (36)
	Reverse	AGGTACTCAGTCATCCACA	
BCL-XL	Forward	AGGATACAGCTGGAGTCAG	Valks et al. (37)
	Reverse	TCTCCTTGTCTACGCTTTCC	
BAK	Forward	CCCAGGACACAGAGGAGGTTT	Zhang et al. (38)
	Reverse	GCCTCCTGTTCCTGCTGATG	
BAX	Forward	ATGGAGCTGCAGAGGATGA	Guo and Li (36)
	Reverse	CCAGTTTGCTAGCAAAGTAG	
Caspase-3	Forward	TGGTGATGAAGGGGTCATTTAT	Lin et al. (39)
	Reverse	TTCGGCTTTCCAGTCAGACTC	
Autophagy	
ATG5	Forward	CACTGGGACTTCTGCTCCTG	Li et al. (40)
	Reverse	TTCTTCAACCAAGCCAAACC	
ATG7	Forward	CAGCAGTGACGATCGGATGA	Liu et al. (41)
	Reverse	TCAAGAACCTGGTGAGGCAC	
Beclin1	Forward	GCCTCTGAAACTGGACACG	Li et al. (40)
	Reverse	CCTCTTCCTCCTGGCTCTCT	
p62	Forward	GGATGACGACTGGACGCATT	Wu et al. (42)
	Reverse	TCTGGTGGGAGATGTGGGTA	
LC3-II	Forward	CCTGCTGCTGGCCGTAGT	Ni et al. (43)
	Reverse	TGATGAAGTCTTCCTGCCAAAA	
Inflammation	
NLRP3	Forward	TGTGAGAAGCAGGTTCTACTCT	Ydens et al. (44)
	Reverse	GGATGCTCCTTGACCAGTTGG	
ASC	Forward	CAGCACAGGCAAGCACTCA	Ydens et al. (44)
	Reverse	GGTGGTCTCTGCACGAACT	
Caspase-1	Forward	GGGACCCTCAAGTTTTGCC	Ydens et al. (44)
	Reverse	GACGTGTACGAGTGGTTGTATT	
IL-1β	Forward	GCAACTGTTCCTGAACTCAACT	Ydens et al. (44)
	Reverse	ATCTTTTGGGGTCCGTCAACT	
IL-18	Forward	GACTCTTGCGTCAACTTCAAGG	Ydens et al. (44)
	Reverse	CAGGCTGTCTTTTGTCAACGA	
(Gapdh)	Forward	TGGACCTCATGGCCTACATG	Kwon et al. (45)
	Reverse	AGGGAGATGCTCAGTGTTGG	

Gapdh (Glyceraldehyde-3-phosphate dehydrogenase) was used as a reference.

### 2.4 Histopathological examination

Spleen tissues were fixed in 10% buffered formalin solution and then embedded in paraffin blocks following routine histological tissue processing steps. Sections of 5 μm thickness were obtained from the paraffin blocks using a rotary microtome. Finally, the obtained sections were deparaffinized and then stained with haematoxylin and eosin (H&E) for histopathological evaluation. Histopathological and immunohistochemical evaluations were performed independently by two blinded observers. All tissue sections were coded prior to analysis to ensure that investigators remained unaware of the experimental group assignments during scoring and quantification.

### 2.5 Immunohistochemical (IHC) staining

Immunohistochemical analyses were performed to evaluate the expression of apoptosis-related proteins (Bcl-2, Bax, cleaved Caspase-3, cleaved Caspase-9), autophagy-associated markers (ATG5, ATG7, Beclin1, LC3A/B, p62), and inflammatory mediators (NLRP3 and cleaved IL-1β) in splenic tissue sections. Paraffin-embedded spleen samples were processed according to a previously established protocol with minor modifications (16). Briefly, sections were deparaffinized in xylene and rehydrated through graded ethanol series. Endogenous peroxidase activity was quenched with 3% hydrogen peroxide for 10 min, followed by washing in phosphate-buffered saline (PBS). Heat-induced antigen retrieval was performed in citrate buffer (pH 6.0) under controlled temperature conditions. Non-specific binding was minimized by incubation with a protein blocking solution for 10 min at room temperature. Sections were incubated overnight at 4 °C with rabbit polyclonal primary antibodies against Bcl-2 (Cat# AF6139), Bax (Cat# AF0120), cleaved Caspase-3 (Asp175; Cat# AF7022), cleaved Caspase-9 (Asp353; Cat# AF5240), ATG5 (Cat# DF6010), ATG7 (Cat# DF6130), Beclin1 (Cat# AF5128), LC3A/B (Cat# AF5402), p62 (Cat# AF6478), NLRP3 (Cat# DF15549), and cleaved IL-1β (Asp116; Cat# AF4006; all from Affinity Biosciences, USA), applied at optimized dilutions according to the manufacturer's recommendations. After washing, sections were incubated with HRP-conjugated goat anti-rabbit IgG (H+L) secondary antibody (polyclonal, Cat# S0001, RRID: AB_2839429, Affinity Biosciences, USA). Immunoreactivity was visualized using a 3,3′-diaminobenzidine (DAB) chromogen substrate system, followed by counterstaining with Mayer's hematoxylin. Stained sections were examined under a light microscope (Olympus BX53, Japan), and digital images were acquired using an Olympus DP27 camera coupled with Olympus cellSens imaging software. For quantitative analysis, three non-overlapping sections per animal were evaluated, and ten randomly selected fields per section were analyzed using ImageJ software: Fiji distribution of ImageJ, U.S. National Institutes of Health, Bethesda, MD, United States. Immunoreactivity was quantified as the percentage of positively stained area (% area). Mean values per animal were calculated and subjected to statistical analysis (19).

### 2.6 Statistics

All statistical analyses were performed using GraphPad Prism software (version 10.5; GraphPad Software Inc., San Diego, CA, USA). Data are presented as mean ± standard error of the mean (SEM), where applicable. For comparisons among the four experimental groups (Cnt, Cd, Prb, and CdPrb), one-way analysis of variance (ANOVA) followed by Tukey's multiple comparisons *post hoc* test was applied to datasets involving group-level comparisons, such as immunohistochemical quantification. For gene expression analyses, relative mRNA levels are presented as log_2_-transformed fold changes (2^−Δ*ΔCt*^) for individual biological replicates without inferential statistical testing. A *p*-value < 0.05 was considered statistically significant. Statistical significance levels in figures are indicated as follows: ^*^p < 0.05, ^**^p ≤ 0.01, ^***^p ≤ 0.001, and ^****^p ≤ 0.0001.

## 3 Results

### 3.1 General experimental outcomes

The effects of the cafeteria diet, SCD Probiotics supplementation, and SCD Probiotics supplementation during the cafeteria diet on body weight, standard chow intake, and cafeteria diet consumption have been comprehensively reported in our previously published studies using the same experimental design and animal cohort and are therefore not repeated here (16).

### 3.2 Apoptosis related mRNA gene expression

**Figure 1:**
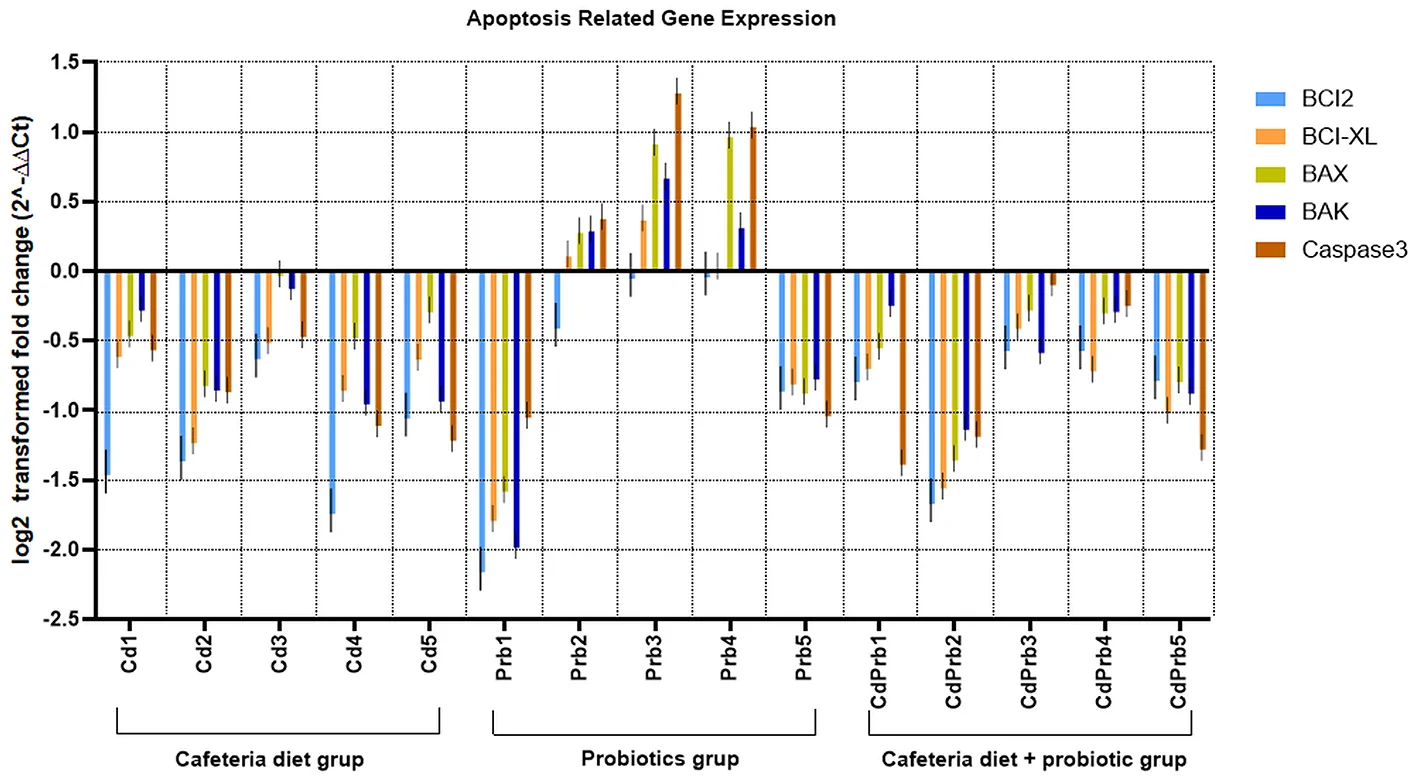
Relative mRNA fold-change patterns of apoptosis-related genes (Bcl-2, Bcl-xL, Bax, Bak, and Caspase-3) in splenic tissue following dietary and probiotic interventions. Expression values were obtained by RT-qPCR using the 2^−Δ*ΔCt*^ method and are shown as log_2_-transformed fold changes relative to the control group. Data are presented descriptively without inferential statistical comparison. Experimental groups: Control (Cnt), cafeteria diet (Cd), probiotic supplementation (Prb), and cafeteria diet plus probiotic (CdPrb).

The expression patterns of apoptosis-related genes, including Bcl-2, Bcl-xL, Bax, Bak, and Caspase-3, were evaluated to explore the effects of dietary stress and probiotic intervention on apoptosis-associated signaling pathways (Figure 1). Cafeteria diet exposure was associated with a fold-change pattern consistent with a shift toward a pro-apoptotic profile. In particular, anti-apoptotic genes (Bcl-2 and Bcl-xL) showed relative decreases, whereas pro-apoptotic markers (Bax, Bak, and Caspase-3) showed relative increases. In contrast, the probiotic-only group displayed fold-change patterns suggestive of a more balanced apoptotic profile, with relative increases in anti-apoptotic genes and relative stabilization or decreases in pro-apoptotic markers. In the cafeteria diet plus probiotic group, the transcript patterns were generally shifted toward attenuation of the cafeteria diet-associated profile, although they did not fully approximate control-level patterns across all markers.

### 3.3 Autophagy related mRNA gene expression

**Figure 2:**
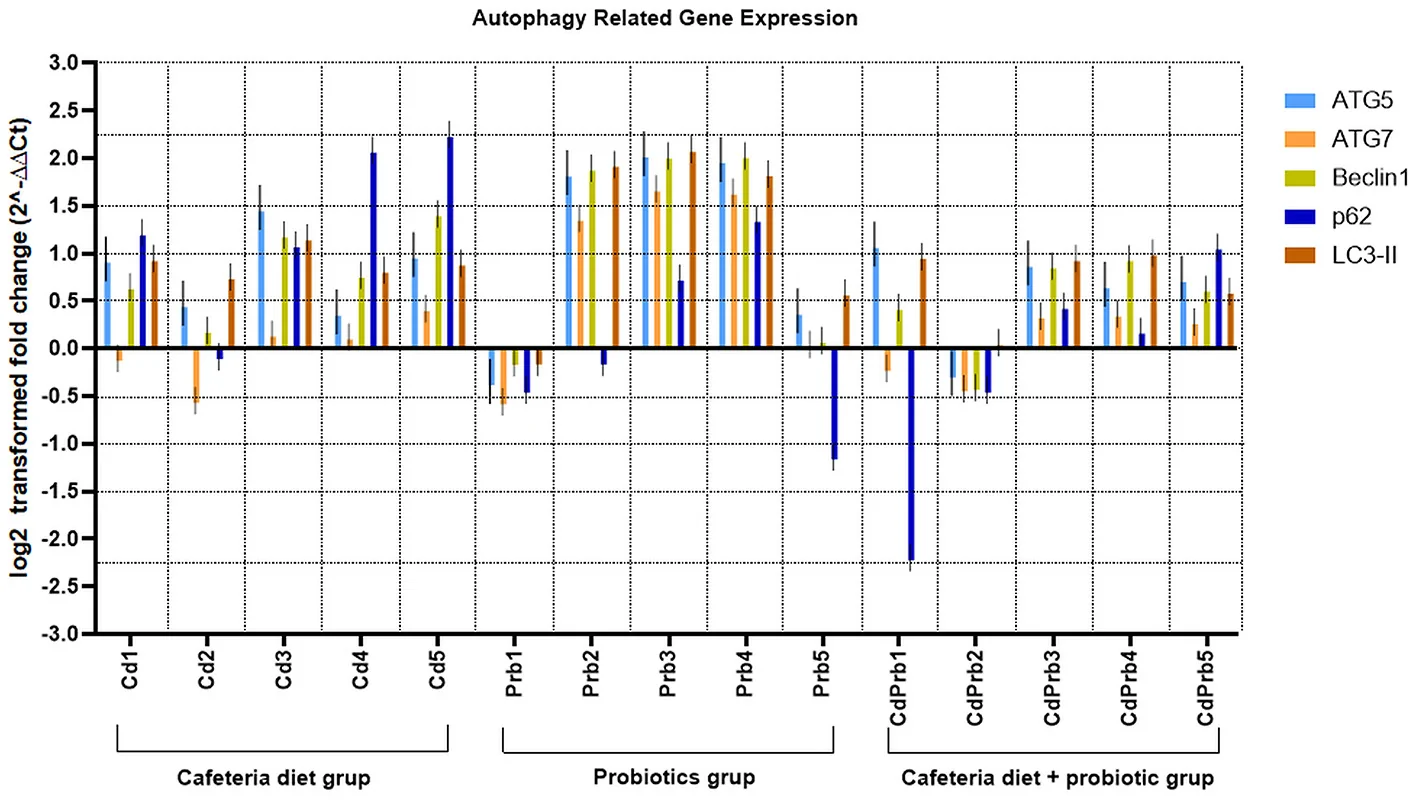
Relative mRNA fold-change patterns of autophagy-related genes (ATG5, ATG7, Beclin-1, LC3-II, and p62) in splenic tissue following dietary and probiotic interventions. Expression values were obtained by RT-qPCR using the 2^−Δ*ΔCt*^ method and are shown as log_2_-transformed fold changes relative to the control group. Data are presented descriptively without inferential statistical comparison. Experimental groups: Control (Cnt), cafeteria diet (Cd), probiotic supplementation (Prb), and cafeteria diet plus probiotic (CdPrb).

The mRNA fold-change patterns of autophagy-related genes, including ATG5, ATG7, Beclin-1, LC3-II, and p62, were evaluated to explore the effects of metabolic stress and probiotic supplementation on autophagy-associated processes (Figure 2). Cafeteria diet exposure was associated with heterogeneous transcript-level changes in autophagy-related markers, with relatively modest alterations in ATG5, ATG7, and Beclin-1. In contrast, p62 showed a relative increase, suggesting a transcript pattern consistent with altered autophagy-associated regulation. The probiotic-only group displayed fold-change patterns characterized by relative increases in core autophagy-related genes (ATG5, ATG7, and Beclin-1) together with a relative decrease in p62. In the combined cafeteria diet and probiotic group, transcript patterns suggested partial attenuation of the cafeteria diet-associated profile, particularly with respect to p62. These findings should be interpreted as descriptive transcript-level patterns rather than direct evidence of altered autophagic flux.

### 3.4 Inflammasome and inflammation related mRNA gene expression

**Figure 3:**
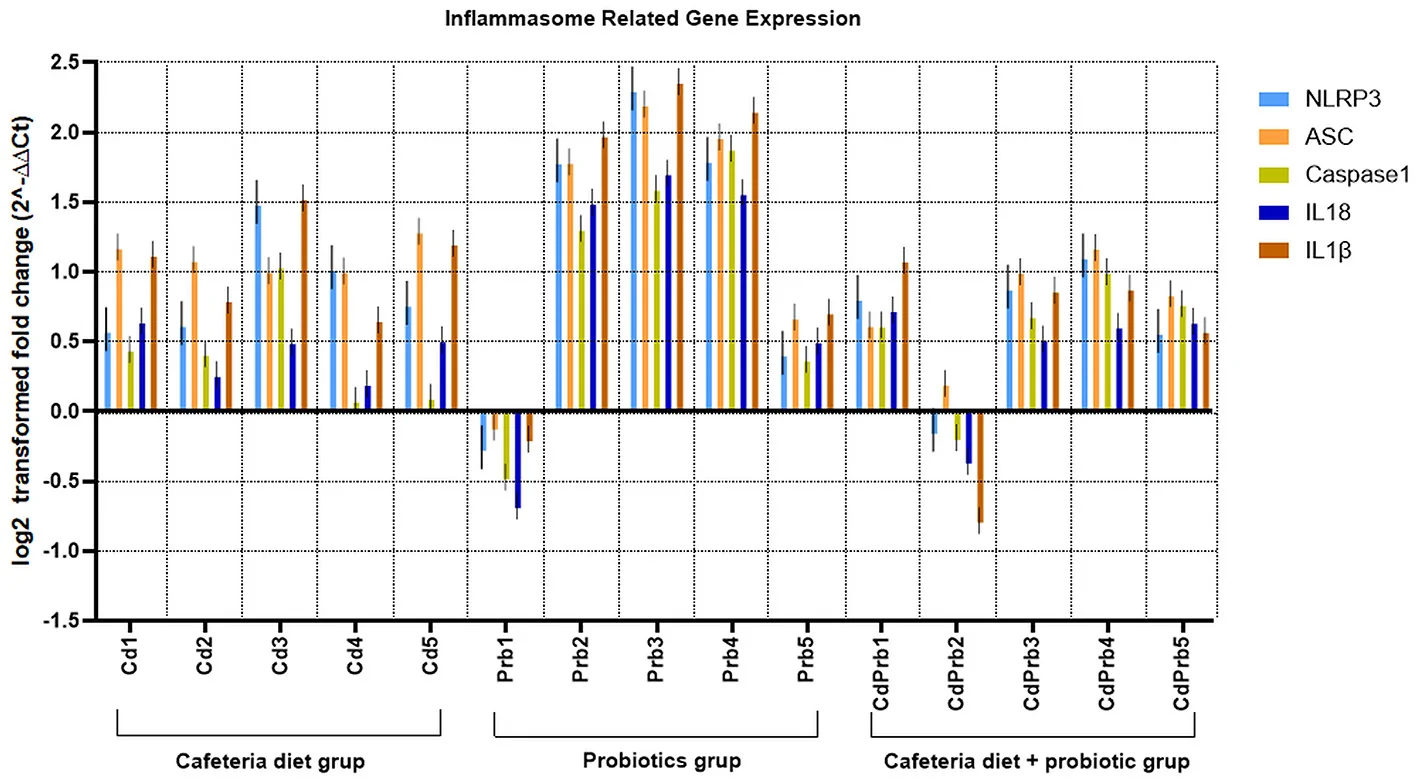
Relative mRNA fold-change patterns of inflammasome-associated genes (NLRP3, ASC, Caspase-1, IL-1β, and IL-18) in splenic tissue following dietary and probiotic interventions. Expression values were obtained by RT-qPCR using the 2^−Δ*ΔCt*^ method and are shown as log_2_-transformed fold changes relative to the control group. Data are presented descriptively without inferential statistical comparison. Experimental groups: Control (Cnt), cafeteria diet (Cd), probiotic supplementation (Prb), and cafeteria diet plus probiotic (CdPrb).

The relative mRNA fold-change patterns of inflammasome-associated genes, including NLRP3, ASC, Caspase-1, IL-1β, and IL-18, were evaluated to assess the effects of dietary intervention and probiotic supplementation on splenic inflammasome-related signaling (Figure 3). Cafeteria diet exposure was associated with a general trend toward higher expression of several inflammasome-related transcripts relative to the control group, although the magnitude of these changes varied across individual markers and samples. Notably, NLRP3 and IL-1β displayed fold-change patterns suggestive of increased inflammasome-associated signaling under high-fat dietary conditions. In the probiotic-only group, several inflammasome-related markers also showed relative increases compared with control patterns, indicating a context-dependent transcriptional response. In the combined cafeteria diet and probiotic group, transcript patterns were generally shifted toward attenuation of the cafeteria diet-associated profile, although this was not consistent across all markers. These transcript-level observations should be interpreted descriptively and do not, by themselves, demonstrate definitive inflammasome activation.

### 3.5 Histopathological examination

**Figure 4:**
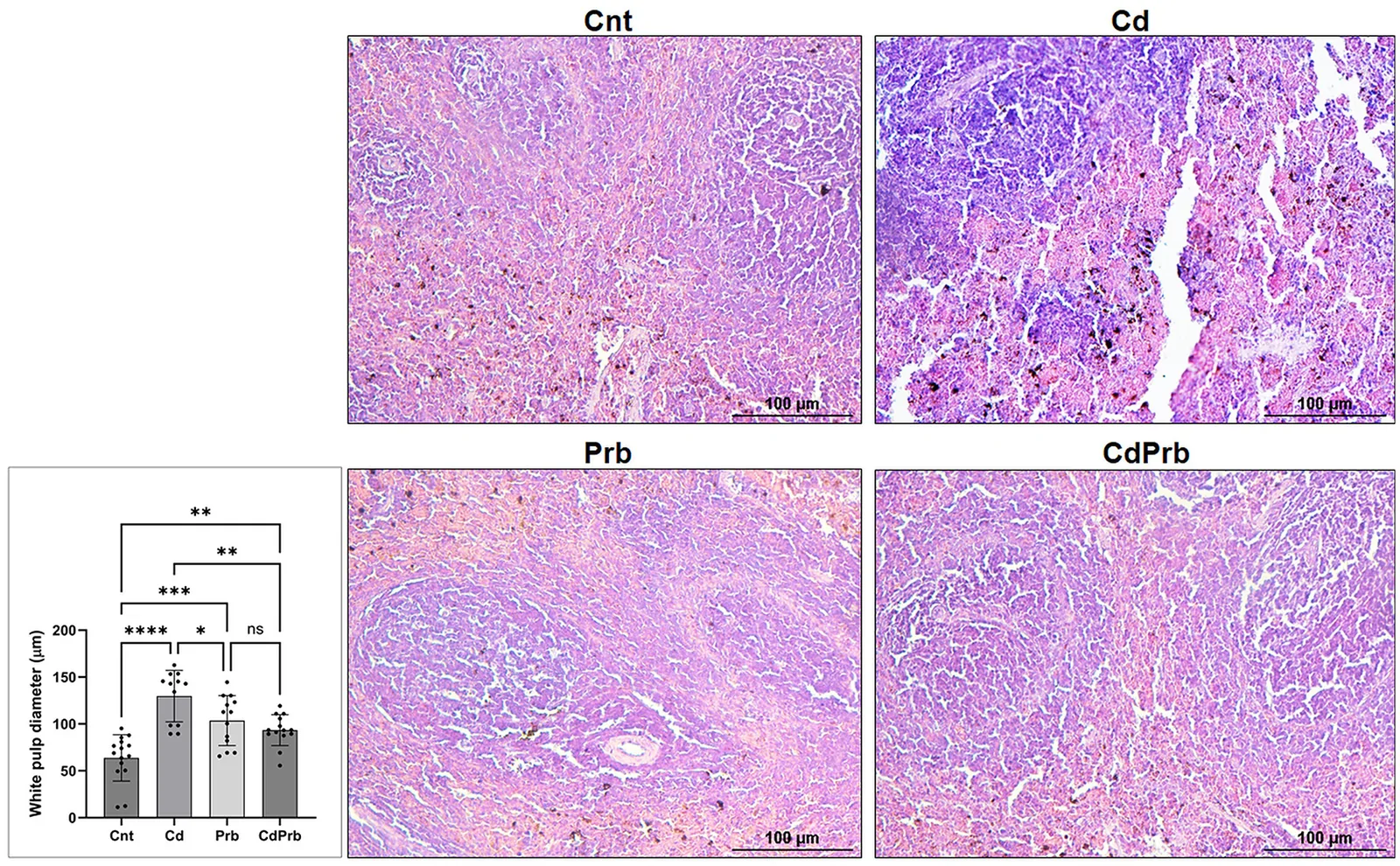
Representative hematoxylin and eosin (H&E)-stained spleen sections from the experi-mental groups: Control (Cnt), Cafeteria diet (Cd), Probiotic (Prb), and Cafeteria diet plus probiotic (CdPrb). Micrographs illustrate representative histological architecture of splenic tissue, with particular emphasis on white pulp structures. Scale bar = 100 μm; magnification × 200. Quantitative analysis of splenic white pulp diameter (μm) is presented for each group. Data are expressed as mean ± SEM (*n* = 6 per group). Statistical significance was determined using one-way ANOVA followed by Tukey's *post hoc* test. Significance levels are indicated as *p < 0.05, **p ≤ 0.01, ***p ≤ 0.001, and ****p ≤ 0.0001.

Histopathological examination revealed significant differences in white pulp diameter among experimental groups (one-way ANOVA, *F* = 17.29, *p* < 0.0001; Figure 4). *Post-hoc* Tukey analysis showed that animals receiving the cafeteria diet exhibited a marked enlargement of white pulp structures compared with the control group (*p* < 0.0001). SCD Probiotics supplementation alone also resulted in significantly greater white pulp diameter relative to controls (*p* = 0.0004). Similarly, the CdPrb group displayed significantly larger white pulp structures compared with the control group (*p* = 0.0092). When treatment groups were compared, the Cd group exhibited significantly greater white pulp diameter than both the Prb group (*p* = 0.0439) and the CdPrb group (*p* = 0.0019). In contrast, no significant difference was observed between the Prb and CdPrb groups (*p* = 0.6896). These findings indicate that the cafeteria diet induces pronounced expansion of splenic white pulp, whereas probiotic supplementation partially attenuates Cd-associated lymphoid enlargement.

### 3.6 Immunohistochemical evaluation of apoptosis markers

**Figure 5:**
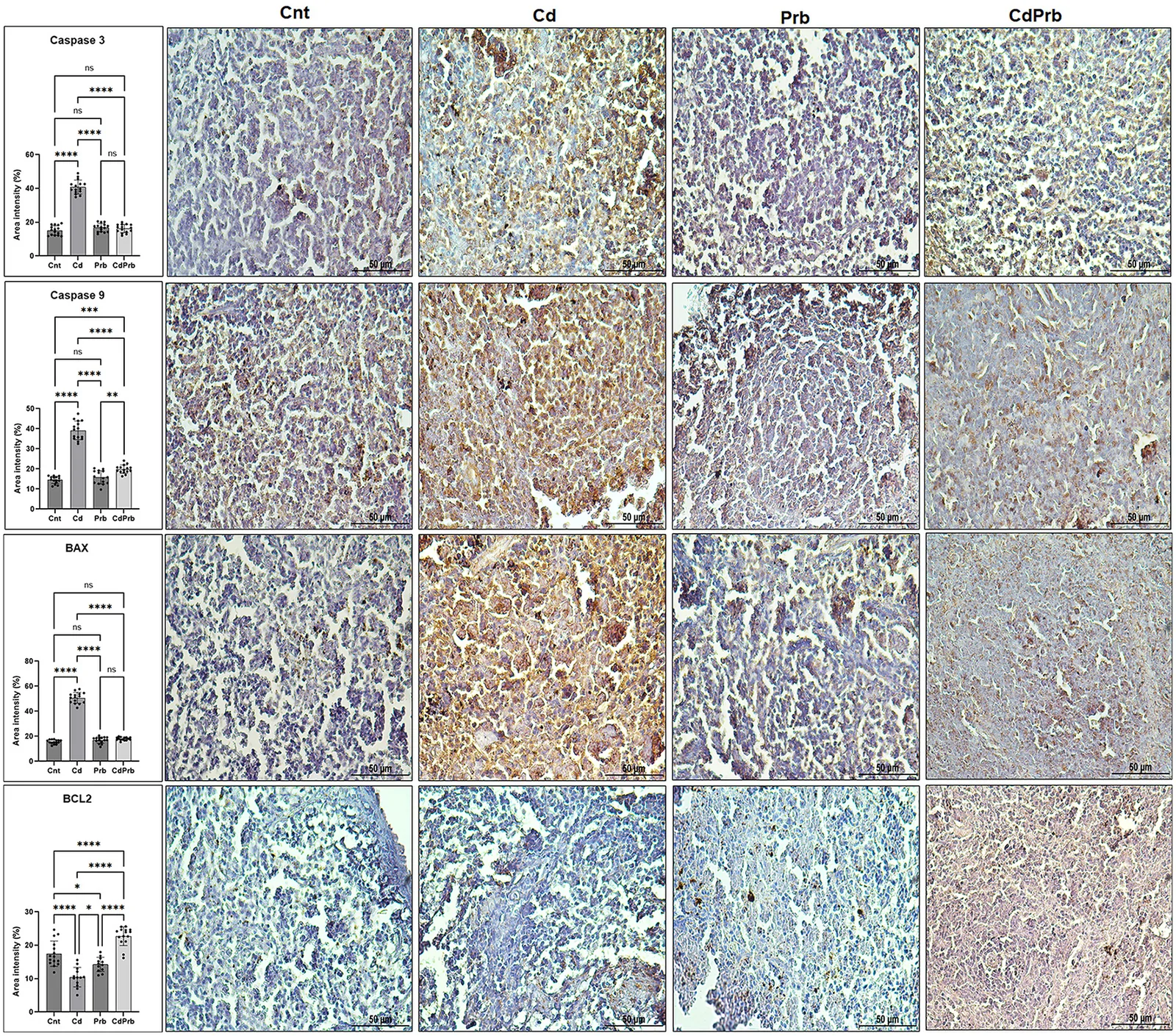
Immunohistochemical evaluation of apoptosis markers in splenic tissue. Representative immunohistochemical staining and quantitative analysis of apoptosis-associated proteins in splenic tissue sections from Control (Cnt), Cafeteria diet (Cd), Probiotic (Prb), and Cafeteria diet plus probiotic (CdPrb) groups. Representative micrographs and quantitative analysis are shown for: Caspase-3, Caspase-9, BAX, and BCL2. Brown staining indicates positive immunoreactivity detected using DAB chromogenic substrate, with hematoxylin counterstaining. Scale bar = 50 μm. Quantification of immunoreactivity was performed using ImageJ software and expressed as per-centage of positively stained area (% area). Data are presented as mean ± SEM. Statistical analysis was conducted using one-way ANOVA with Tukey's *post hoc* test. ***p < 0.001, ****p < 0.0001; ns, not significant.

Immunohistochemical analysis demonstrated significant group-dependent differences in apoptotic protein expression across Cnt, Cd, Prb, and CdPrb groups (one-way ANOVA, all *p* < 0.0001; Figure 5). Caspase-3 showed a strong overall effect (*F* = 249.0, *p* < 0.0001). Cd exhibited significantly higher immunoreactivity compared with Cnt (*p* < 0.0001). Both Prb and CdPrb displayed significantly lower staining intensity than Cd (*p* < 0.0001 for both) and did not differ from Cnt. No significant difference was observed be-tween Prb and CdPrb. Caspase-9 expression also differed significantly among groups (*F* = 196.0, *p* < 0.0001). Cd increased Caspase-9 immunoreactivity relative to Cnt (p < 0.0001). CdPrb was significantly lower than Cd (*p* < 0.0001) but remained higher than Cnt (*p* = 0.0002). Prb did not differ from Cnt but differed significantly from Cd (*p* < 0.0001). BAX demonstrated a marked group effect (*F* = 591.9, *p* < 0.0001). Cd significantly increased BAX immunoreactivity compared with Cnt (*p* < 0.0001). Both Prb and CdPrb exhibited significantly lower expression than Cd (*p* < 0.0001 for both) and did not differ from Cnt. BCL2 showed a distinct pattern (*F* = 41.36, *p* < 0.0001). Cd significantly reduced BCL2 staining relative to Cnt (*p* < 0.0001). CdPrb exhibited significantly higher BCL2 immu-noreactivity than both Cd (*p* < 0.0001) and Cnt (*p* < 0.0001). Prb also showed higher BCL2 expression compared with Cd (*p* < 0.05). Overall, immunohistochemical findings con-firmed significant group-dependent modulation of splenic apoptosis-related protein ex-pression under developmental Cd exposure and probiotic intervention.

### 3.7 Immunohistochemical evaluation of autophagy markers

**Figure 6:**
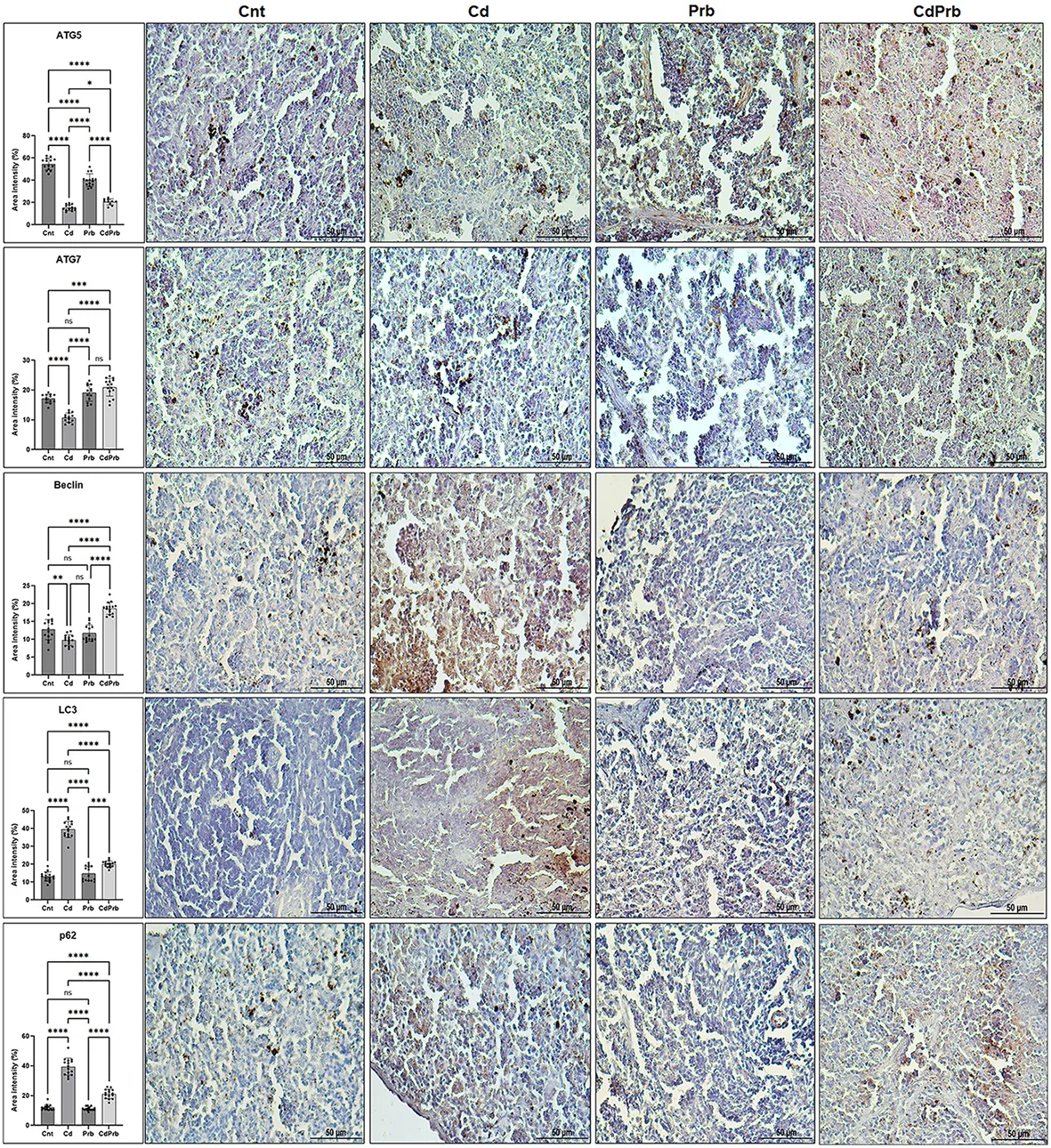
Immunohistochemical analysis of autophagy-related protein expression in splenic tissue. Representative immunohistochemical staining and quantitative analysis of autophagy markers in spleen sections from experimental groups (Cnt, Cd, Prb, CdPrb). Protein expression was evaluated for: ATG5, ATG7, Beclin1, LC3, and p62. Positive immunoreactivity was visualized using DAB chromogenic detection with hematoxylin counterstaining. Scale bar = 50 μm. Quantitative analysis of immunostaining intensity was performed using ImageJ software and expressed as percentage of positively stained area (% area). Data represent mean ± SEM. Statistical comparisons were performed using one-way ANOVA followed by Tukey's *post hoc* test. *p < 0.05, **p < 0.01, ***p < 0.001, ****p < 0.0001; ns, not significant.

Immunohistochemical analysis revealed significant group-dependent differences in autophagy-related protein expression across Cnt, Cd, Prb, and CdPrb groups (one-way ANOVA, all *p* < 0.0001; Figure 6). ATG5 demonstrated a strong overall group effect (*F* = 222.2, *p* < 0.0001). Cd significantly reduced ATG5 immunoreactivity compared with Cnt (*p* < 0.0001). Both Prb and CdPrb showed significantly higher ATG5 staining than Cd (*p* < 0.0001 and *p* = 0.0354, respectively). However, CdPrb remained lower than Cnt (*p* < 0.0001). ATG7 expression also differed significantly among groups (*F* = 48.39, *p* < 0.0001). Cd reduced ATG7 staining relative to Cnt (*p* < 0.0001). Both Prb and CdPrb exhibited higher expression than Cd (*p* < 0.0001 for both), although CdPrb remained lower than Cnt (*p* = 0.0005). Beclin1 showed a significant group effect (*F* = 48.05, *p* < 0.0001). Cd reduced Beclin1 immunoreactivity compared with Cnt (*p* = 0.0021). CdPrb exhibited higher staining than both Cnt (*p* < 0.0001) and Cd (*p* < 0.0001). Prb did not differ from Cnt. LC3-II demonstrated a strong overall group effect (*F* = 188.9, *p* < 0.0001). Cd increased LC3-II immunoreactivity relative to Cnt (*p* < 0.0001). Both Prb and CdPrb showed significantly lower LC3-II expression compared with Cd (*p* < 0.0001 for both). CdPrb remained higher than Cnt (*p* < 0.0001). p62 also exhibited a significant group effect (*F* = 218.8, *p* < 0.0001). Cd increased p62 accumulation compared with Cnt (*p* < 0.0001). Both Prb and CdPrb demonstrated significantly lower p62 staining than Cd (*p* < 0.0001 for both). However, CdPrb remained higher than Cnt (*p* < 0.0001).

### 3.8 Immunohistochemical evaluation of inflammasome markers

**Figure 7:**
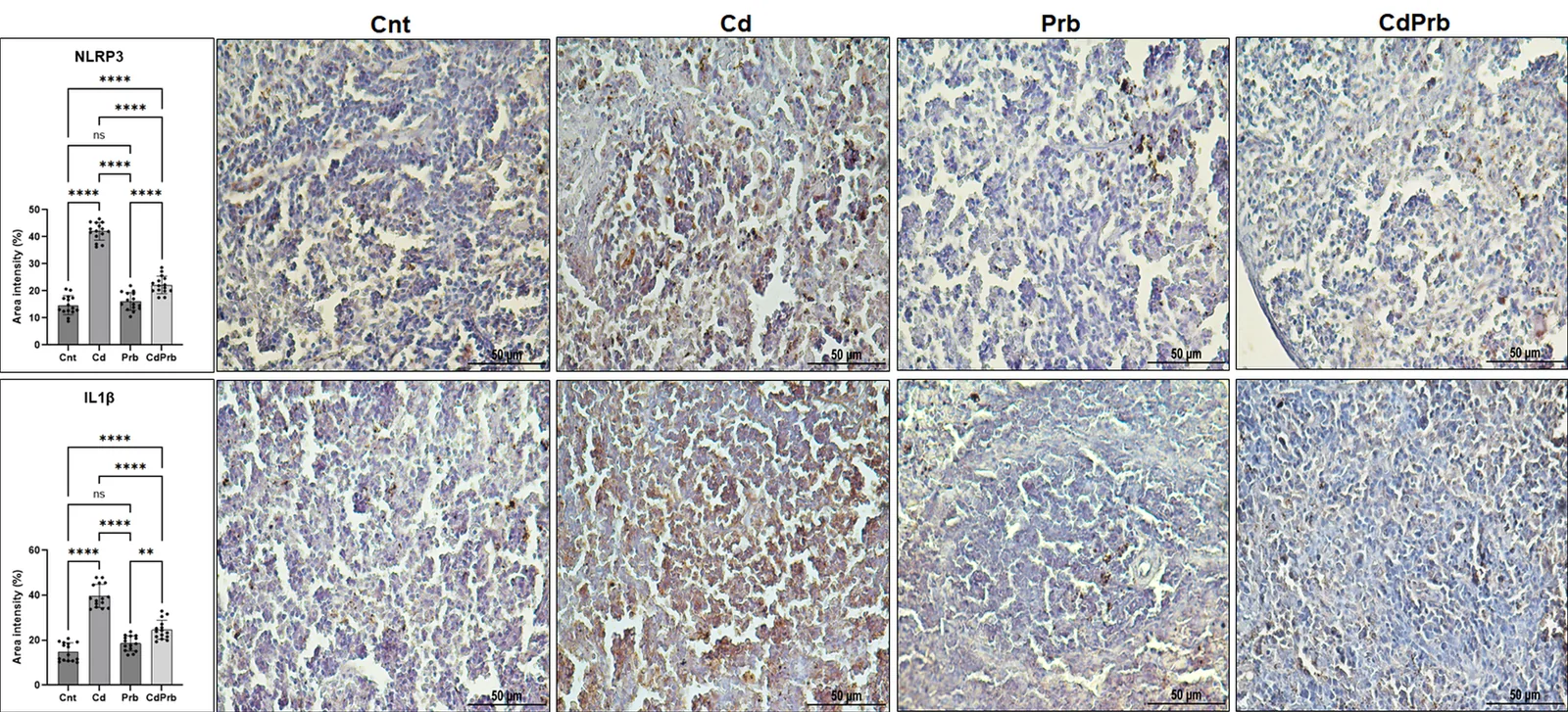
Probiotic supplementation attenuates cafeteria diet–induced inflammasome activation in splenic tissue. Representative immunohistochemical staining and quantitative analysis of inflammasome-associated proteins in splenic tissue. Protein expression of NLRP3 and IL-1β was assessed in Control (Cnt), Cafeteria diet (Cd), Probiotic (Prb), and Cafeteria diet plus probiotic (CdPrb) groups. Positive staining was visualized using DAB chromogen and hematoxylin counterstaining. Scale bar = 50 μm. Quantitative analysis of immunoreactivity was performed using ImageJ software and expressed as percentage of positive staining area (% area). Values represent mean ± SEM. Statistical analysis was performed using one-way ANOVA with Tukey's multiple comparison test. **p < 0.01, ***p < 0.001, ****p < 0.0001; ns, not significant.

Immunohistochemical analysis demonstrated significant group-dependent differences in inflammasome-related protein expression across Cnt, Cd, Prb, and CdPrb groups (one-way ANOVA, all *p* < 0.0001; Figure 7). NLRP3 exhibited a strong overall group effect (*F* = 213.7, *p* < 0.0001). Cd significantly increased NLRP3 immunoreactivity compared with Cnt (*p* < 0.0001). Prb did not differ from Cnt. CdPrb showed significantly lower NLRP3 expression than Cd (*p* < 0.0001), but remained higher than Cnt (*p* < 0.0001). CdPrb also differed significantly from Prb (*p* < 0.0001). IL-1β also demonstrated a significant group effect (*F* = 99.22, *p* < 0.0001). Cd increased IL-1β immunoreactivity relative to Cnt (*p* < 0.0001). Prb did not differ from Cnt. CdPrb exhibited significantly lower IL-1β expression compared with Cd (*p* < 0.0001), yet remained higher than Cnt (*p* < 0.0001). CdPrb differed significantly from Prb (*p* = 0.0014). Overall, immunohistochemical findings demonstrated significant group dependent modulation of splenic inflammasome related protein expression under developmental Cd exposure and probiotic intervention.

## 4 Discussion

The present study provides integrated molecular and histopathological evidence that developmental exposure to a high-fat cafeteria diet is associated with alterations in splenic apoptotic, autophagy-associated, and inflammasome-related signaling. Concurrent SCD probiotic supplementation partially modulated several of these diet-associated changes. These findings identify the spleen as a responsive immunometabolic organ in early-life dietary programming and suggest that gut-targeted microbial interventions may influence systemic immune stress responses extending beyond the intestine.

### 4.1 Apoptosis

Cafeteria diet exposure shifted splenic tissue toward a pro-apoptotic state, as demonstrated by altered gene expression and immunohistochemical findings. The intrinsic mitochondrial pathway is highly sensitive to metabolic stress, and increased Bax expression together with reduced Bcl-2 levels is widely recognized as a hallmark of apoptosis activation under obesogenic conditions (8, 20). Consistent with this framework, high-fat diet models have reported similar shifts in Bax/Bcl-2 balance accompanied by caspase-3 activation, supporting a mechanistic link between metabolic overload and intrinsic apoptotic signaling (21, 22).

The pro-apoptotic pattern observed in splenic tissue aligns with systemic oxidative and inflammatory alterations previously documented in other organs within the same developmental model. In earlier analyses, cafeteria diet exposure impaired hepatic antioxidant defense systems and increased oxidative stress burden, effects attenuated by SCD Probiotics supplementation (15). Intestinal structural disruption and elevated pro-inflammatory cytokines (TNF-α, IL-1β) were likewise observed in ileum and colon tissues (16). Metagenomic profiling from the same cohort further demonstrated reduced microbial diversity and altered short-chain fatty acid (SCFA)–associated pathways, including disturbances in acetate, propionate, and butyrate metabolism (23). Given the role of microbiota-derived metabolites in immune homeostasis, reduced SCFA availability may contribute to altered apoptotic regulation within splenic immune cells. Because SCD Probiotics is a multi-strain formulation containing both bacterial species and yeast, the present data do not permit attribution of the observed effects to any single strain or metabolite; therefore, the findings should be interpreted as formulation-specific rather than strain-specific.

Probiotic supplementation attenuated the pro-apoptotic signature observed in CAF-fed animals. Experimental studies have shown that selected Lactobacillus strains can modulate Bax/Bcl-2 expression and suppress caspase-3 activation under metabolic stress, while Bifidobacterium species may indirectly influence apoptotic priming through attenuation of inflammatory signaling (24, 25). These documented strain-specific properties provide biological plausibility for the normalization of splenic apoptotic markers observed following probiotics supplementation in the present study.

Collectively, these findings indicate that developmental exposure to a cafeteria diet induces apoptotic priming in splenic tissue, while microbiota-targeted intervention may partially restore apoptotic balance within the immune compartment.

### 4.2 Autophagy

Cafeteria diet exposure altered ATG5, ATG7, Beclin-1, LC3, and p62 expression, suggesting dysregulated autophagic flux rather than simple suppression. In particular, the marked increase in p62 expression observed in the cafeteria diet group indicates impaired autophagic degradation rather than enhanced autophagy, reflecting disruption of autophagic flux. High-fat diets induce compensatory autophagy followed by impaired autophagosome–lysosome fusion and defective mitophagy (26). Lipid overload disrupts lysosomal function and mitochondrial turnover, amplifying oxidative stress and inflammatory signaling.

The splenic autophagy pattern observed here mirrors similar dysregulation described in hepatic and other metabolic tissues under obesogenic stress. SCFAs particularly butyrate modulate autophagic pathways through AMPK activation and mTOR suppression (27). Microbiota derived metabolites, particularly SCFAs, are established modulators of cellular metabolic signaling pathways that intersect with autophagy regulation. SCFAs have been shown to influence AMPK–mTOR signaling and cellular energy homeostasis, processes that are tightly linked to autophagy-associated responses under metabolic stress (28). In the metagenomic and metabolomic analyses conducted in parallel, probiotic supplementation partially restored SCFA-associated pathways, suggesting that normalization of microbial metabolite profiles may have contributed to the modulation of autophagy-associated gene expression observed in splenic tissue.

Probiotic supplementation partially normalized autophagy-related markers. This normalization was particularly evident in the reduction of p62 levels, suggesting restoration of autophagic flux rather than mere transcriptional modulation of autophagy-related genes. Prior work demonstrated that SCD Probiotics modulate hepatic biomolecular structure and attenuate inflammasome activation in aged liver models (17). In intermittent fasting paradigms, probiotic intervention improved hepatic biomolecule dynamics and reduced fibrosis while influencing autophagy-associated signatures (29). Multi-strain probiotic formulations have been reported to modulate metabolic stress–associated signaling pathways in diet-induced obesity models. In particular, certain *Lactiplantibacillus plantarum* strains have been shown to influence AMPK-related metabolic signaling and oxidative stress adaptation under high-fat diet conditions, providing biological plausibility for autophagy-associated modulation in the present model (30, 31).

Restoration of the autophagy–apoptosis balance in splenic tissue likely represents a central mechanism by which probiotics preserve immune homeostasis during developmental metabolic stress. Autophagic competence is a determinant of immune cell survival and inflammatory threshold under metabolic challenge, supporting the systemic relevance of the regulatory effects observed here.

### 4.3 Inflammasome activation

Cafeteria diet was associated with increased NLRP3 inflammasome-related signaling in splenic tissue, as evidenced by increased gene expression and immunoreactivity. NLRP3 is widely recognized as a metabolic sensor responsive to lipid excess and mitochondrial stress signals under obesogenic conditions (13, 32). Experimental and immunometabolic studies indicate that metabolic overload lowers the activation threshold for inflammasome assembly in immune cells, thereby amplifying inflammatory responsiveness.

Autophagy has been shown to negatively regulate inflammasome activation by limiting the accumulation of cellular stress signals (33). Disruption of autophagy-associated regulation, as observed in the present model, may therefore contribute to enhanced inflammasome-associated inflammatory signaling. Caspase-1–dependent processing of pro-inflammatory cytokines further propagates inflammatory signaling once NLRP3 is engaged (34).

Notably, probiotic supplementation did not exert a uniformly suppressive effect on inflammasome activation. In the probiotic-only group, increased expression of certain inflammasome-related markers was observed, suggesting that probiotic administration alone may induce a context-dependent immunomodulatory response rather than direct suppression. The relative reduction of NLRP3 expression and downstream cytokines in the probiotic-treated cafeteria diet group, compared to the cafeteria diet group alone, indicates attenuation of excessive inflammasome-associated signaling under metabolic stress conditions rather than complete inhibition. This finding is consistent with previous observations demonstrating reduced NLRP3 gene expression and immunoreactivity following SCD Probiotics administration in aged liver models (17), as well as decreased IL-1β and TNF-α expression in intestinal tissue under cafeteria diet exposure (16).

Metagenomic analyses conducted in parallel revealed restoration of microbial diversity and normalization of short-chain fatty acid–associated metabolic pathways (23). SCFAs, particularly butyrate, have been reported to suppress NLRP3 activation through HDAC-dependent mechanisms and modulation of cellular metabolic signaling (13, 35). Although mitochondrial ROS and ion flux were not directly assessed in the present study, normalization of microbiota-derived metabolites may contribute to reduced inflammatory priming and attenuated inflammasome responsiveness in splenic tissue.

Collectively, convergent findings across gut, liver, microbiome, and spleen support a model in which probiotic supplementation modulates systemic inflammatory tone. Rather than acting as a direct suppressor of inflammasome-related signaling, probiotic intervention appears to reshape inflammasome responsiveness in a context-dependent manner under metabolic stress conditions. Rather than invoking direct mitochondrial restoration, the present data support attenuation of inflammasome-associated signaling within the broader context of developmental metabolic stress.

### 4.4 Limitations

Several limitations should be acknowledged. Autophagic flux was inferred from marker expression and immunoreactivity rather than dynamic lysosomal inhibition assays. Autophagic flux was inferred from RT-qPCR and immunohistochemical marker profiles rather than directly demonstrated by immunoblot-based or lysosomal inhibition assays. Therefore, interpretations related to autophagic flux should be considered descriptive and hypothesis-generating. Functional immune profiling of splenic cell subsets was not performed, limiting cell-specific mechanistic resolution. Although microbiome and SCFA analyses were conducted in the same experimental cohort, direct correlation modeling between microbial taxa, metabolite abundance, and splenic molecular markers was not undertaken. Finally, only male animals were included, restricting evaluation of sex-dependent immunometabolic responses. Future investigations integrating multi-omics correlation analysis, immune cell phenotyping, and functional inflammasome inhibition approaches would further refine the mechanistic framework proposed herein. Moreover, because the study relied on RT-qPCR and immunohistochemical marker profiles without pathway-specific functional interventions, the present data do not permit causal inference regarding crosstalk among apoptosis, autophagy, and inflammasome-related signaling pathways.

## 5 Conclusion

In conclusion, developmental exposure to a cafeteria diet was associated with marked alterations in splenic apoptotic, autophagy-associated, and inflammasome-related markers. SCD probiotic supplementation partially attenuated several of these diet-associated changes. Together with the histopathological and immunohistochemical findings, these results support the spleen as a responsive immunometabolic organ during early-life nutritional stress. These findings extend the current understanding of early-life nutritional programming and support a potential modulatory role of this multi-strain probiotic formulation in splenic immunometabolic stress. However, the present data do not establish causal crosstalk among these pathways.

## Data Availability

The data presented in the study are included in the article/Supplementary material. Raw qPCR Ct values and related source data are provided in the Supplementary material.
